# Fluid Intelligence and Cognitive Reflection in a Strategic Environment: Evidence from Dominance-Solvable Games

**DOI:** 10.3389/fpsyg.2016.01188

**Published:** 2016-08-10

**Authors:** Nobuyuki Hanaki, Nicolas Jacquemet, Stéphane Luchini, Adam Zylbersztejn

**Affiliations:** ^1^Université Côte d'Azur, Centre National de la Recherche Scientifique, GREDEGValbonne, France; ^2^CES, Paris School of Economics and University Paris 1 Panthéon-SorbonneParis, France; ^3^Aix-Marseille University (Aix-Marseille School of Economics), Centre National de la Recherche Scientifique and EHESSMarseille, France; ^4^Univ Lyon, Université Lumière Lyon 2, GATE L-SE UMR 5824Ecully, France

**Keywords:** dominance solvability, cognitive skills, CRT, Raven's test, experiment, C72, D83

## Abstract

Dominance solvability is one of the most straightforward solution concepts in game theory. It is based on two principles: dominance (according to which players always use their dominant strategy) and iterated dominance (according to which players always act as if others apply the principle of dominance). However, existing experimental evidence questions the empirical accuracy of dominance solvability. In this study, we study the relationships between the key facets of dominance solvability and two cognitive skills, cognitive reflection, and fluid intelligence. We provide evidence that the behaviors in accordance with dominance and one-step iterated dominance are both predicted by one's fluid intelligence rather than cognitive reflection. Individual cognitive skills, however, only explain a small fraction of the observed failure of dominance solvability. The accuracy of theoretical predictions on strategic decision making thus not only depends on individual cognitive characteristics, but also, perhaps more importantly, on the decision making environment itself.

## 1. Introduction

Consider a game in which every decision maker is faced with a finite set of choices such that one specific choice always brings him higher monetary payoff than other choices, irrespective of the choices made by other players. In this situation, the individual choice boils down to going for either a higher or a lower monetary payoff. The straightforward response of a decision maker who cares about his monetary payoff is to disregard dominated actions—i.e., actions that may only deteriorate payoff relative to other actions. This dominance principle is the most basic solution concept of game theory (Camerer, 2003). It becomes very powerful when embedded in a strategic reasoning as a stepwise process. In each step, the dominance principle implies that dominated strategies should be eliminated from an agent's strategy space. In an important class of games—known as dominance-solvable games—this iterated elimination of dominated strategies leads to a unique solution.

Strikingly, the data collected from numerous experiments on dominance-solvable games raise important questions about the empirical accuracy of predictions derived from this principle. Subjects tend to display less strategic sophistication than is needed to justify many applications of iterated dominance (and related refinements) to model human decision making in strategic environments (Crawford, 2004). The beauty contest game is one of the textbook examples of this issue^1^. A given set of players is asked to choose a number in the range [0, 100]. To win the game, a player should choose a number that is the closest to *p* = 2/3 of the average of all chosen numbers. Any number above 2/3 × 100 ≈ 66.7 violates first-order dominance, because the average has to be lower than 100. Knowing this, players should all choose numbers no greater than 66.7, meaning that their average may not exceed 2/3 × 66.7 ≈ 44.5. This reasoning lowers the target as the number of iterations increases, eventually leading to the unique Nash equilibrium in which all players choose 0. In many experimental studies of this game, the numbers chosen by players are used as a proxy of the depth of iterated reasoning.^2^ A well replicated stylized fact is to observe 1/3 of subjects choosing a number higher than 67, and at least 1/3—a number between 44 and 67.

This paper focuses on one of the earliest and simplest example of such an empirical inaccuracy of dominance solvability, adapted from a 2 × 2 game discussed in Rosenthal (1981) and first brought to the laboratory by Beard and Beil (1994)^3^. The normal-form representation of this game is given in Table 1. With *L* < *S* < *H*, *m* < *h*, and *s* < *h*, the game is one-step dominance solvable: the elimination of player B's weakly dominated strategy *l* immediately leads to the Pareto-Nash equilibrium (*R, r*)^4^.

**Table 1:** **Generic form of the normal representation of Rosenthal (1981) dominance solvable game**.

		**Player B**	
		****l****	****r****
**Player A**	*L*	(S; s)	(S; s)
	*R*	(L; m)	(H; h)

In line with observed behavior in other dominance solvable games, numerous studies (summarized in Table 2) find frequent failures to achieve the Pareto-Nash equilibrium. In spite of variations in the design (described in the table), deviations from the standard theoretical predictions are systematic and sizable. First, dominance is frequently violated by player Bs. Depending on the exact experimental setup, up to 27% column players choose a strictly dominated action. Second, player As violate iterated dominance, even in those cases in which player Bs commonly obey dominance. As an example, while only 6% of player Bs violate dominance in Jacquemet and Zylbersztejn (2014)-ET2 and BT2, 26% of row players still contradict the predictions of dominance solvability by choosing *L* (and this figure may even attain 86% in other instances, see Beard, Beil – Tr. 5 in Table 2). As shown in the three middle columns of the table, both the absolute and the relative size of the stakes vary a great deal from one study to the other. Several lessons emerge from this accumulated evidence. First, both players react to their own monetary incentives. Second, in some cases player As also adjust their behavior to player Bs' incentives. Finally, as shown by Jacquemet and Zylbersztejn (2014), players' inefficient behavior does not fade away with repetition and cannot be explained by inequality aversion (as framed by Fehr and Schmidt, 1999).

**Table 2:** **Overview of existing experimental evidence**.

**Experiment**	**Form**	**Payoff**			**Outcomes (%)**				
		***(L)***	***(R,r)***	***(R,l)***	****L****	****R, r****	****R, l****	****r|R****	****r****
Beard, Beil–Tr.1	Seq	(9.75; 3.0)	(10; 5.0)	(3; 4.75)	66	29	6	83	—
Beard, Beil–Tr.2	Seq	(9.00; 3.0)	(10; 5.0)	(3; 4.75)	65	35	0	100	—
Beard, Beil–Tr.3	Seq	(7.00; 3.0)	(10; 5.0)	(3; 4.75)	20	80	0	100	—
Beard, Beil–Tr.4	Seq	(9.75; 3.0)	(10; 5.0)	(3; 3.00)	47	53	0	100	—
Beard, Beil–Tr.5	Seq	(9.75; 6.0)	(10; 5.0)	(3; 3.00)	86	14	0	100	—
Beard, Beil–Tr.7	Seq	(58.50; 18.0)	(18.0; 28.50)	(60; 30.0)	67	33	0	100	—
Beard et al.–Tr.1	Seq	(1450; 450)	(1500; 750)	(450; 700)	79	18	3	83	—
Beard et al.–Tr.2	Seq	(1050; 450)	(1500; 750)	(450; 700)	50	32	18	64	—
Goeree, Holt–Tr.1	Ext	(80; 50)	(90; 70)	(20; 10)	16	84	0	100	—
Goeree, Holt–Tr.2	Ext	(80; 50)	(90; 70)	(20; 68)	52	36	12	75	—
Goeree, Holt–Tr.3	Ext	(400; 250)	(450; 350)	(100; 348)	80	16	4	80	—
Cooper, Van Huyck–Tr.9	Str	(4; 1)	(6; 5)	(2; 4)	27	—	—	—	86
Cooper, Van Huyck–Tr.9	Ext	(4; 1)	(6; 5)	(2; 4)	21	—	—	—	84
JZ, 2014–BT1	Str	(9.75; 3.0)	(3.0; 4.75)	(10; 5.0)	51	41	8	84	81
JZ, 2014–ET1	Str	(9.75; 5.0)	(5.0; 9.75)	(10; 10.0)	54	33	13	72	73
JZ, 2014–ET3	Str	(9.75; 5.5)	(5.5; 8.50)	(10; 10.0)	39	48	13	79	76
JZ, 2014–ET4	Str	(8.50; 5.5)	(5.5; 8.50)	(10; 10.0)	25	61	14	82	82
JZ, 2014–ET2	Str	(8.50; 8.5)	(6.5; 8.50)	(10; 10.0)	26	70	4	94	94
JZ, 2014–BT2	Str	(8.50; 7.0)	(6.5; 7.00)	(10; 8.5)	26	70	4	94	94

*For each implementation in row, the first column describes the actual design of the experiment: simultaneous-move strategic-form game (Str), simultaneous-move extensive-form game (Ext), sequential-move game (Seq). The monetary payoffsof each outcome, displayed in columns 2–4, are in USD in Beard and Beil (1994) and Cooper and Van Huyck (2003), in cents of USD in Goeree and Holt (2001), in Yens in Beard et al. (2001), and in Euros in Jacquemet and Zylbersztejn (2014). The game is repeated ten times in changing pairs in Jacquemet and Zylbersztejn (2014), and one-shot in all other instances*.

The aim of the present paper is to explore whether this empirical puzzle is related to players' cognitive skills. In this sense, our investigation belongs to a recent and growing body of experimental studies in both psychology and economics which investigate the relationship between strategic behavior and cognitive skills^5^. The main conclusion that can be drawn from these studies is that high cognitive skills predict strategic sophistication and efficient decision making. First, people with high cognitive skills make more accurate predictions about other people's intentions. Recent evidence from psychological research reveals the relationship between cognitive skills and the theory of mind. Using the “Reading the Mind in the Eyes” test (RMET, Baron-Cohen et al., 2001) to measure one's theory of mind, Ibanez et al. (2013) find that people with higher cognitive skills are better at infering the internal emotional states of others^6^. Relatedly, the results of a neuroeconomic experiment on the *p*-beauty contest game by Coricelli and Nagel (2009) suggest that strategic thinking about other players' thoughts and behavior is implemented by medial prefrontal cortex (mPFC) – one of the brain areas commonly associated with theory of mind^7^. An economic experiment by Carpenter et al. (2013) also shows that people with higher cognitive ability make more accurate predictions of others' choices in a 20-player beauty contest game. Second, people with higher cognitive skills apply more sophisticated reasoning and are more apt in strategic adaptation. Burks et al. (2009) report that subjects with higher cognitive skills more accurately predict others' behavior in a sequential prisoners' dilemma game, and adapt their own behavior more strongly. In the context of the *p*-beauty contest game, subjects with higher cognitive skills are not only found to carry out more steps of reasoning on the equilibrium path (Burnham et al., 2009; Brañas-Garza et al., 2012), but also to adapt their behavior to their opponents' cognitive skills (Gill and Prowse, forthcoming) as well as to their beliefs about their opponents' cognitive skills (Fehr and Huck, 2015). Third, cognitive skills may be associated with the economic efficiency of outcomes of both individual and group activities. Corgnet et al. (2015b) find that higher cognitive skills predict better performance and less shirking in an experimental labor task (summing up tables of 36 numbers without using a pen). Jones (2008), Al-Ubaydli et al. (in press), and Proto et al. (2014) report that groups with higher cognitive skills attain higher cooperation rates in repeated prisoner's dilemma games. On the other hand, Al-Ubaydli et al. (2013) do not find a relationship between group members' average cognitive skills and the efficiency of outcomes in a stag hunt coordination^8^.

Our contribution is two-fold. First, we provide new evidence on the relationship between strategic behavior and cognitive skills. We show that systematic mismatches between theoretical predictions and actual behavior in a classic 2 × 2 dominance-solvable game have cognitive underpinnings. Subjects with higher cognitive skills are found to be more likely to play dominant strategy and to best respond to other's strategy. Furthermore, cognitive skills predict strategic sophistication: only those players with sufficiently high cognitive ability are found to display sensitivity to the presence of uncertainty about others' behavior. Our second contribution lies in experimental methodology. We extend the recent body of laboratory experiments comparing the performance of different measures of cognitive skills in predicting economic behavior. Notwithstanding the previous results (see e.g., Brañas-Garza et al., 2012; Corgnet et al., 2015a), we report that the Raven's test score is a more general predictor of strategic behavior than the Cognitive Reflection Test score.

## 2. Experimental design

Our experiment is based on a 2 × 2 factorial design that varies the payoff matrix and the nature of player B. Each of the four resulting experimental treatments is implemented through a between-subject procedure—each subject participates in only one experimental condition. This data come from a large dataset, part of which has been previously used by Hanaki et al. (2016). The main focus of that study is player As' behavior under strategic uncertainty and its relation to monetary incentives and fluid intelligence. Certain elements of their design (such as the use of Human and Robot conditions and interest in players' cognitive skills) inevitably needed to be adopted in the present study in order to address a much more general question of the empirical validity of the solution concept of dominance solvability. More precisely, we are interested in both players' behavior (so as to measure the use of dominance by player Bs and the use of iterated dominance by player As under different information structures). We also make a methodological contribution, since in this paper we associate players' behavior with multiple facets of cognitive skills: fluid intelligence (measured by Raven's test) and cognitive reflection (measured by CRT).

Our first treatment variable is the size of the stakes, as represented by Game 1 and Game 2 in Table 3. Although they have the same strategic properties, these two game matrices differ in terms of the saliency of monetary incentives to use (iterated) dominance. In Game 2, player As may earn a surplus of only 0.25 when moving from *L* to (*R, r*) (with payoff going from 9.75 to 10), while ending up in (*R, l*) is relatively costly (yielding only 3). In Game 1, the potential gains and losses from action *R* relative to *L* are more balanced: the gain from moving from *L* to (*R, r*) increases to 1.5 (with payoff moving from 8.5 to 10), while the outcome (*R, l*) becomes less costly (now yielding 6.5). The incentives of player Bs, in turn, go in the opposite direction: the gain from using the dominant strategy *r* (and conditional on player As' choice *R*) is lower in Game 1 [with payoff increasing from 4.75 to 5 between (*R, l*) and (*R, r*)] than in Game 2 (where payoff increases from 8.5 to 10). In line with Jacquemet and Zylbersztejn (2014) and Hanaki et al. (2016) (who report that both players only react to their own monetary incentives) and as discussed in Section 3.1, each of these games generates sizable yet diverse empirical violations of dominance solvability. These two games together thus provide a wide range of monetary incentives to use dominance solvability within a common strategic environment^9^.

**Table 3:** **The experimental games**.

**GAME 1**			
		**B**	
		****l****	****r****
**A**	*L*	(8.50 ; 3.00)	(8.50 ; 3.00)
	*R*	(6.50 ; 4.75)	(10.00 ; 5.00)
**GAME 2**			
		**B**	
		****l****	****r****
**A**	*L*	(9.75 ; 8.50)	(9.75 ; 8.50)
	*R*	(3.00 ; 8.50)	(10.00 ; 10.00)

Our second treatment variable is related to the nature of player B (the column player) who may be represented either by a human subject (Human condition) or a pre-programmed computer (Robot condition). The Human condition enables us to capture two cardinal breaches of dominance solvability: the failure to use the dominant strategy (player Bs' behavior) and the failure to best respond to others' dominant actions (player As' behavior). However, the latter behavior occurs under strategic uncertainty and thus might stem from two distinct sources: bounded rationality and rational behavior under uncertainty. More precisely, player As may simply have a limited capability of best responding to dominant strategy, but may also intentionally refrain from best responding when in doubt about player Bs' use of dominant strategy. To separate these two effects, we introduce the Robot condition in which a human subject acting as player A interacts with a computerized player B who is pre-programmed to always choose *r*. We clearly inform the subjects in the Robot condition that they are interacting with a pre-programmed computer: “**the computer chooses** *r* **at each round, without exception**” (bold in the original instruction sheet). This is the only difference in the rules and procedures between Human and Robot conditions^10^. Thus, the key property of the Robot condition as compared to the Human condition is neutralizing strategic uncertainty player As face, while maintaining space for boundedly rational behavior.

The design of the experiment is otherwise the same in all four experimental conditions. We explore whether behavior is sensitive to learning by considering ten uniform, one-shot interactions. In order to homogenize incentives across rounds, the following rules are implemented: all games are played in strict anonymity, roles are fixed, and subjects' payoffs are computed based one randomly drawn round. In the Human condition, players are matched into pairs using a perfect stranger, round-robin scheme, which guarantees that subjects are involved in a series of one-shot interactions despite the repetition of the game^11^.

Our control variables also include two measures of cognitive skills. Both of them are introduced as part of a post-experimental supplementary task. Subjects' participation is rewarded with extra five Euros; otherwise, their answers are not incentivized^12^. The supplementary task starts with a debriefing question, where subjects are asked to “report any information they find relevant about how their decisions has been made.” Then, we implement the following measures of cognitive skills.

The first task is the standard Cognitive Reflection Test based on Frederick (2005) which “*measures cognitive reflectiveness or impulsiveness, respondents' automatic response versus more elaborate and deliberative thought*” (Brañas-Garza et al., 2012, p. 255). It contains three questions:

1. A notebook and a pencil cost 1.10 Euros in total. The notebook costs 1 Euro more than the pencil. How much does the pencil cost?
2. If it takes 5 machines 5 min to make 5 widgets, how long would it take 100 machines to make 100 widgets?
3. In a lake, there is a patch of lily pads. Every day, the patch doubles in size. If it takes 48 days for the patch to cover the entire lake, how long would it take for the patch to cover half of the lake?

Subjects are informed that this set of three questions should be answered within 30 s (although we allow them to provide answers even after this time has elapsed). In this way, subjects can be classified according to their overall score (that is, the total number of correct answers) which can range from 0 to 3.

The second task is Raven's progressive matrix test (often called Raven's test), a picture based, non-verbal measure of fluid intelligence, that is “*the capacity to think logically, analyze and solve novel problems, independent of background knowledge*” (Mullainathan and Shafir, 2013, p. 48). It is widely used by, e.g., psychologists, educators, and the military (Raven, 2000). It consists of a series of tasks to be solved within a fixed amount of time. In each task, a subject should pick a single element (among eight options) that best fits a set of eight pictures. The level of difficulty increases from one question to the other^13^. In our experiment, each participant is given a series of 16 tasks to be solved within 10 min. Individual scores in Raven' test are computed as the number of correct answers to the 16 items of the test.

### 2.1. Experimental procedures

For each game matrix, we run three Human sessions (involving 20 subjects per session: 10 player As interacting with 10 player Bs), and two Robot sessions (involving 20 player As per session interacting with automated player Bs). Subjects are given a fixed fee equal to five euros to compensate participation to the experiment.

Upon arrival to the laboratory, participants are randomly assigned to their computers and asked to fill in a short administrative questionnaire containing basic questions about their age, gender, education, etc. Experimental instructions are then read aloud: subjects are informed that they will play multiple rounds of the same game, each round with a different partner, and that their own role will remain unchanged throughout the experiment. Finally, subjects are asked to answer a short comprehension quiz. Once the quiz and any questions from participants are answered, the experiment begins. After each of the ten rounds of the game, subjects are only informed of their own payoffs. Information about past choices and payoffs is updated after each round and displayed at the bottom of the screen. Take-home earnings correspond to the outcome of a single round that is randomly drawn at the end of each experimental session.

In addition, the experimental game is followed by supplementary tasks. An additional five euros fee is paid to each subject for completing this part. Immediately after the end of the experimental game, participants are provided with a brief round-by-round summary of their decisions and outcomes, and are asked to provide in a blank space on their computer screens any relevant comments in particular about what might have affected their decisions during the experiment. Subjects are also asked to solve the CRT test and a reduced-form Raven's test described above.

All the sessions were conducted in February and March 2014. Out of the 200 participants (94 males), 155 were students with various fields of specialization. The majority of subjects (65%) had already taken part in economic experiments. Participants' average age was 25.6 (st. dev. is 7.5). All sessions took place at the *Laboratoire d'Economie Experimentale de Paris* (LEEP) at Paris School of Economics. Subjects were recruited via an on-line registration system based on Orsee (Greiner, 2015) and the experiment was computerized through software developed under Regate (Zeiliger, 2000) and z-Tree (Fischbacher, 2007). Sessions lasted about 45–60 min, with an average payoff of roughly 18.83 euros (including a five euros show-up fee and five euros for completing the post-experimental tasks).

## 3. Results

Our main experimental results can be summarized as follows. First, in line with the existing literature, we observe systematic and sizable deviations from standard predictions based on the principle of dominance solvability. This phenomenon persists across game matrices and despite repetition. Second, we associate strategic behavior with cognitive skills. We find that Raven's test score is a more reliable predictor of strategic behavior than CRT score: whenever the latter predicts behavior, the former does too, but not *vice versa*. Subjects with higher Raven's test scores are more likely to use the dominant strategy and to best respond to other player's dominant strategy. Unlike those with low Raven's test score, they also react to the presence of strategic uncertainty.

### 3.1. Aggregate behavior in experimental games

Table 4 outlines the main patterns of behavior in our experimental games. The statistical significance of the changes observed in this table is tested by Models 1–3 in Table 5. We first focus on the aggregate frequency of Pareto-Nash equilibrium (*R, r*) – the sole outcome that survives the iterated elimination of (weakly) dominated strategies—found in the Human condition. In both games, we observe substantial deviations from the predictions of this solution concept: overall, players attain the (*R, r*) outcome 58% of times in Game 1 and 43% in Game 2 (Model 1, *H*_0_ : β_1_ = 0, *p* = 0.318). We also observe that efficiency increases over time: in both games, we observe the lowest frequency of (*R, r*) in the initial round (0.333 in Game 1 and 0.200 in Game 2), whereas the highest frequency of (*R, r*) occurs in the final round (0.700 in Game 1 and 0.533 in Game 2).

**Table 4:** **Aggregate results**.

	**Round**										**Overall**
	**1**	**2**	**3**	**4**	**5**	**6**	**7**	**8**	**9**	**10**	
***Pr*** **(*****R,r*****) in the Human condition**											
Game 1	0.333	0.600	0.667	0.700	0.567	0.600	0.433	0.633	0.567	0.700	0.580
Game 2	0.200	0.333	0.400	0.400	0.433	0.500	0.500	0.433	0.467	0.533	0.420
***Pr*** **(*****r*****) by player B in the Human condition**											
Game 1	0.767	0.800	0.867	0.900	0.800	0.800	0.700	0.833	0.867	0.800	0.813
Game 2	0.833	0.933	0.900	0.933	1.000	0.933	0.933	0.900	0.900	0.933	0.920
***Pr*** **(*****R*****) by player A in the Human condition**											
Game 1	0.500	0.733	0.700	0.767	0.767	0.800	0.700	0.767	0.700	0.867	0.730
Game 2	0.300	0.333	0.400	0.400	0.433	0.533	0.533	0.500	0.500	0.533	0.447
***Pr*** **(*****R*****) by player A in the Robot condition**											
Game 1	0.700	0.750	0.750	0.725	0.800	0.800	0.800	0.825	0.800	0.775	0.773
Game 2	0.500	0.575	0.725	0.575	0.800	0.700	0.700	0.775	0.775	0.775	0.690

*Columns 1–10 summarize the frequencies of outcomes (defined in rows) as % of all outcomes observed in each round of a given experimental treatment. The last column provides overall results*.

**Table 5:** **Aggregate results: statistical support**.

	**Model 1**	**Model 2**	**Model 3**
	***Pr* (*R, r*)**	***Pr* (*r*)**	***Pr* (*R*)**
Constant (β_0_)	0.580^***^	0.813^***^	0.730^***^
	(0.144)	(0.032)	(0.084)
1[*Game* 2] (β_1_)	–0.160	0.107^*^	–0.283^**^
	(0.103)	(0.044)	(0.140)
1[*Robot*] (β_2_)			0.043
			(0.103)
1[*Robot*] × 1[*Game* 2] (β_3_)			0.201
			(0.161)
*N*	600	600	1400
*R*^2^	0.026	0.025	0.066

Estimates of linear probability models on outcome (R, r) (Model 1), decision r by player B (Model 2) and decision R by player A (Model 3). Standard errors (in parentheses) are clustered at the session level in Human treatments (three clusters per game matrix, six in total) and individual level in the Robot condition (40 clusters per game matrix, 80 in total) and computed using the delete-one jackknife procedure. All models contain a dummy variable set to 1 for game matrix 2 (and 0 for game matrix 1). In Model 3, we also introduce an additional dummy variable set to 1 for Robot condition (and 0 for Human condition) and well as the interaction between these two variables.

*/**/*** *indicate significance at the 10/5/1% level*.

To further explore the roots of these deviations, we turn to the aggregate patterns of both players' behavior in Human and Robot conditions. We focus on three behavioral dimensions of dominance solvability: the use of dominant strategy (captured by player Bs' behavior in the Human condition) and the ability to best respond to other player's dominant action with and without bearing the uncertainty about the latter (which is captured by player As' behavior in the Human and Robot conditions, respectively).

Inefficiency is caused by both players, although their roles differ from one game to another: the scope of inefficient behavior is similar for both players in Game 1, and highly asymmetric in Game 2. Overall, player As select action *R* with probability 0.730 in Game 1 and 0.447 in Game 2 (Model 3, *H*_0_ : β_1_ = 0, *p* = 0.047). However, player As' behavior happens to be misaligned with player Bs' actual decisions which follow the opposite trend: the total frequency of action *r* increases from 0.813 in Game 1 to 0.920 in Game 2 (Model 2, *H*_0_ : β_1_ = 0, *p* = 0.060). Importantly, the data from Robot sessions suggest that the uncertainty about player Bs' behavior is not the only driver of player As' choices. Player As frequently and systematically fail to best respond to player Bs' dominant action even when the latter comes with certainty in the Robot condition, although their willingness to select action *R* increases in both games as compared to the Human condition (to 0.773 in Game 1 and 0.690 in Game 2)^14^. The fact that inefficient actions from player As prevail in the absence of strategic uncertainty may suggest that at least some of them are boundedly rational decision makers.

In the next section, we analyze how these three behavioral components of dominance solvability vary as a function of players' cognitive skills.

### 3.2. Cognitive skills and strategic behavior

The average score in Raven's test (CRT) is 8.679 out of 16 with SD 3.117 (0.479 out of 3 with SD 0.852). Our experimental sample is properly randomized across treatments regarding both measures. We do not reject the null hypothesis that Raven's test scores have the same distributions in all treatments (*p* = 0.275, Kruskal-Wallis test). A Kruskal-Wallis test applied to the CRT scores leads to the same conclusion (*p* = 0.502).

We also replicate several results from previous studies combining Raven's test and CRT regarding the relationship between both scores as well as gender differences (Brañas-Garza et al., 2012; Corgnet et al., 2015a). There is a moderate, yet highly significant correlation between Raven and CRT scores (Spearman's ρ = 0.306, *p* < 0.001) which suggests that they may have a common source, but do not capture the same cognitive skills. Furthermore, the average score of males is significantly higher than the average score of females (Raven's test: 9.382 with SD 0.341 vs. 8.014 with SD 0.384, *p* = 0.009; CRT: 0.676 with SD 0.111 vs. 0.291 with SD 0.087, *p* = 0.007; two-sided *t*-tests)^15^.

We also observe that many subjects (70%) of our 200 participants fail to provide at least one correct answer in our standard CRT. 16% provide exactly one, 8% – two, and 6% – three correct answers. This stands in line with Brañas-Garza et al. (2012) who report the respective frequencies of 67, 23, 9, and 1% for a similar sample size (*N* = 191), and echoes the scores in the least performant sample reported in a seminal study by Frederick (2005): out of 138 students of the University of Toledo, 64% provide no correct answer, 21% provide one, 10% provide two, and 5% provide three corrects answers.

#### 3.2.1. Cognitive predictors of strategic behavior: aggregate results

**Figure 1:**
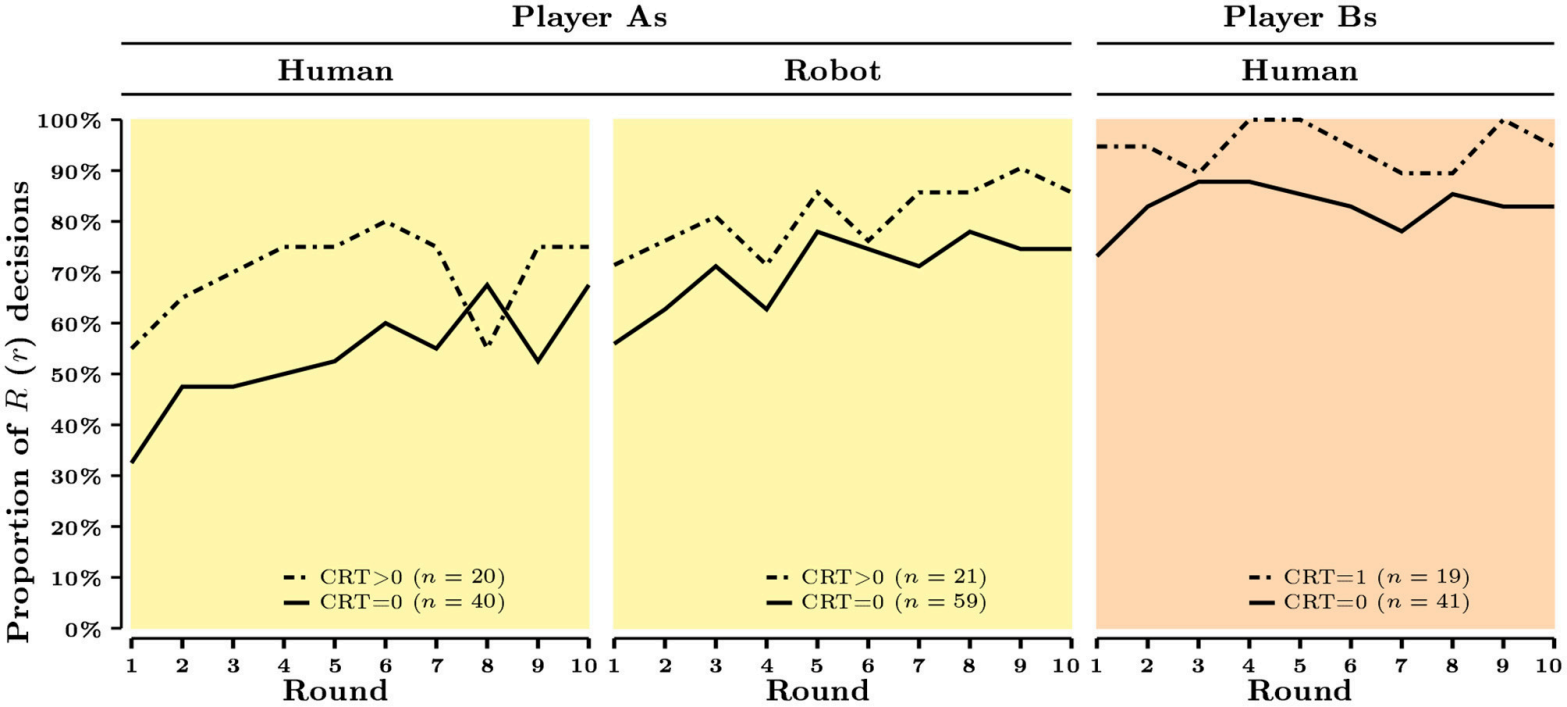
**CRT score and aggregate behavior across rounds and treatments**.

**Figure 2:**
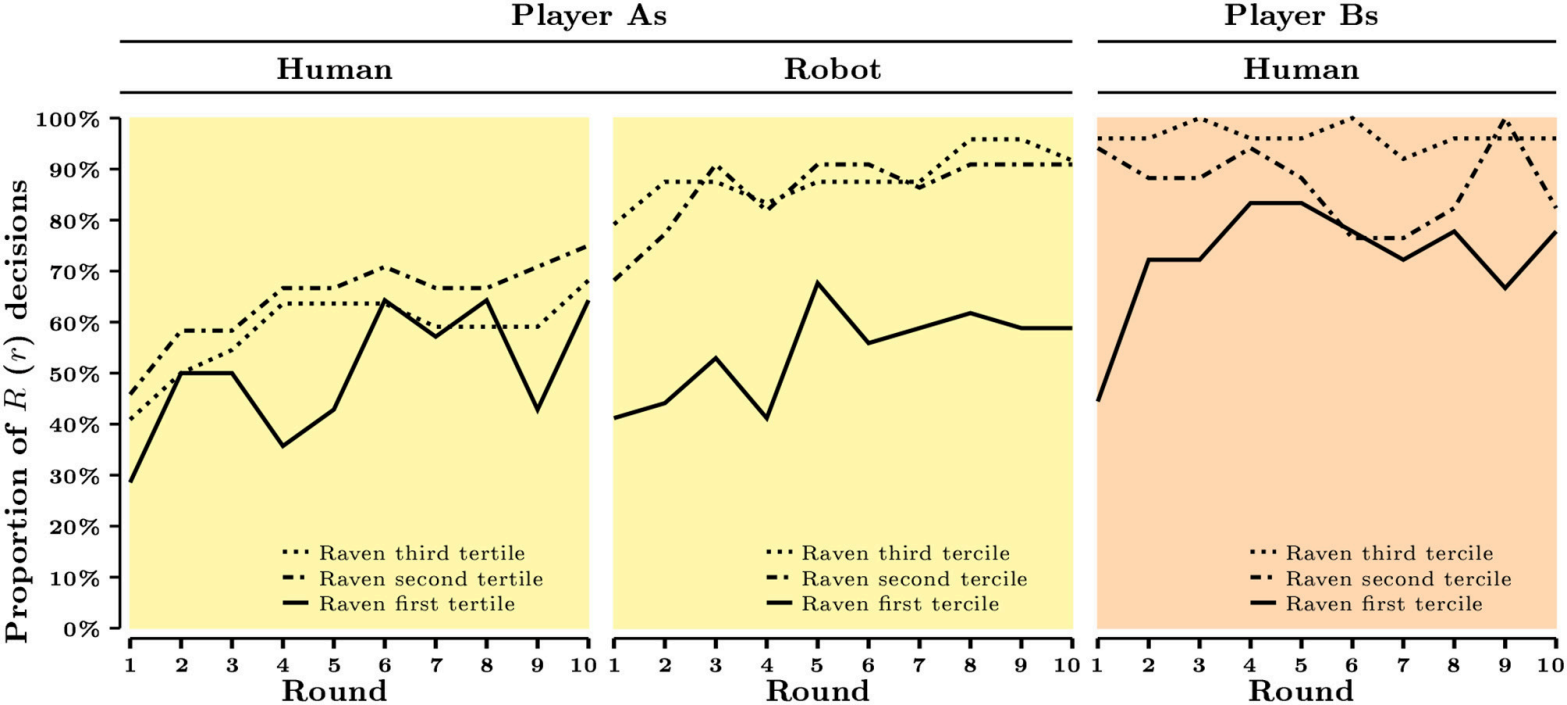
**Raven's test score and aggregate behavior across rounds and treatments**.

In this part, we study the cognitive correlates of strategic behavior. Figures 1, 2 present the aggregate evolution of behavior as a function of cognitive skills, measured either by CRT score or by Raven's test score across roles (player A or player B) and experimental conditions (Human or Robot).

In Figure 1, the sample is divided into two subsamples: subjects who provided at least one correct answer to CRT (referred to as *CRT* > 0) and those who did not (referred to as *CRT* = 0). The aggregate patterns of behavior weakly differ between the two subsamples. Bootstrap proportion tests fail to reject the null hypothesis that the overall proportions of decision *R* are the same for both CRT categories in the Human condition (*p* = 0.126) and in the Robot condition (*p* = 0.235)^16^. The aggregate proportions of decision *r*, in turn, are found to be statistically different (*p* = 0.037), subjects with a CRT score zero being less likely to play *r* than subjects who gave at least one correct answer.

In Figure 2, we split our sample into three subsamples based on Raven's test score (1st tertile: less than 8 correct answers, 2nd tertile: between 8 and 10 correct answers, 3rd tertile: more than 10 correct answers). Although, bootstrap proportion tests suggest that player As' behavior in the Human condition does not vary significantly between these three subsamples (1st tertile vs. 2nd tertile: *p* = 0.255, 2nd vs. 3rd: *p* = 0.580, 1st vs. 3rd: *p* = 0.565), significant differences arise for both player As in the Robot condition (*p* = 0.001, *p* = 0.735, *p* < 0.001, respectively) and for player Bs (*p* = 0.064, *p* = 0.057, *p* < 0.001, respectively). Raven's test score seems to have a more systematic association with players' behavior than CRT score, although both measures fail to predict behavior under strategic uncertainty.

#### 3.2.2. Cognitive skills and dominance solvability: regression analysis

In what follows, we provide further econometric insights into these preliminary results. Following Brañas-Garza et al. (2012); Corgnet et al. (2015a), we use three individual characteristics discussed in the previous section – gender, Raven's test score and CRT score (kept as a dummy variable with value 1 if the subject gave at least one correct answer at the CRT test and 0 otherwise) – to explain behavior in our experimental games^17^. The econometric specification is based on the linear probability model and the estimation procedure is outlined in Jacquemet and Zylbersztejn (2014). We also control for payoff scheme and repetition effects by including game matrix and round dummies. We consider three different outcome variables: player As' behavior in the Human and the Robot treatment, and player Bs' behavior in the Human treatment. Given the correlation between CRT and Raven's test scores, including both variables in the model might result in multicollinearity and lead to the under-rejection of the nullity of respective coefficients. For each outcome, we first include these two measures separately in Models 1 and 2, while Model 3 includes both variables. This evidence is summarized in Table 6.

**Table 6:** **Cognitive predictors of strategic behavior: regression analysis**.

	******Pr****** **(********R********) by player A**						**Pr (********r********) by player B**		
	**Human condition**			**Robot condition**			**Human condition**		
	**Model 1**	**Model 2**	**Model 3**	**Model 1**	**Model 2**	**Model 3**	**Model 1**	**Model 2**	**Model 3**
Const.	0.423^***^	0.552^**^	0.563^**^	0.573^***^	0.242^*^	0.240^*^	0.705^***^	0.430^***^	0.444^***^
	(0.080)	(0.176)	(0.197)	(0.088)	(0.135)	(0.135)	(0.027)	(0.103)	(0.099)
1[CRT>0]	0.131		0.152	0.062		(0.024)	0.109^*^		0.046
	(0.095)		(0.121)	(0.102)		(0.102)	(0.047)		(0.036)
Raven		0.013	0.018		0.0426^***^	0.0434^***^		0.0313^**^	0.0287^**^
		(0.025)	(0.027)		(0.013)	(0.012)		(0.009)	(0.008)
1[*Game* 2]	−0.270^*^	0.263	0.266	0.068	0.054	0.056	0.100	0.132^*^	0.129^*^
	(0.129)	(0.139)	(0.136)	(0.083)	(0.076)	(0.079)	(0.052)	(0.056)	(0.056)
1[Male]	0.132	0.187	0.158	0.096	0.072	0.077	0.025	0.024	0.017
	(0.126)	(0.100)	(0.107)	(0.090)	(0.076)	(0.089)	(0.046)	(0.046)	(0.047)
Round:									
2	0.133	0.133	0.133	0.063	0.063	0.063	0.067	0.067	0.067
	(0.092)	(0.092)	(0.092)	(0.048)	(0.048)	(0.048)	(0.042)	(0.042)	(0.042)
3	0.150	0.150	0.150	0.138^***^	0.138^***^	0.138^***^	0.083	0.083	0.083
	(0.109)	(0.109)	(0.109)	(0.050)	(0.050)	(0.050)	(0.048)	(0.048)	(0.048)
4	0.183^**^	0.183^**^	0.183^**^	0.050	0.050	0.050	0.117^*^	0.117^*^	0.117^*^
	(0.070)	(0.070)	(0.070)	(0.047)	(0.047)	(0.047)	(0.048)	(0.048)	(0.048)
5	0.200^*^	0.200^*^	0.200^*^	0.200^***^	0.200^***^	0.200^***^	0.100	0.100	0.100
	(0.089)	(0.089)	(0.089)	(0.045)	(0.045)	(0.045)	(0.052)	(0.052)	(0.052)
6	0.267^**^	0.267^**^	0.267^**^	0.150^***^	0.150^***^	0.150^***^	0.067	0.067	0.067
	(0.088)	(0.088)	(0.088)	(0.054)	(0.054)	(0.054)	(0.049)	(0.049)	(0.049)
7	0.217^*^	0.217^*^	0.217^*^	0.150^***^	0.150^***^	0.150^***^	0.017	0.017	0.017
	(0.098)	(0.098)	(0.098)	(0.047)	(0.047)	(0.047)	(0.048)	(0.048)	(0.048)
8	0.233^**^	0.233^**^	0.233^**^	0.200^***^	0.200^***^	0.200^***^	0.067	0.067	0.067
	(0.088)	(0.088)	(0.088)	(0.057)	(0.057)	(0.057)	(0.056)	(0.056)	(0.056)
9	0.200	0.200	0.200	0.188^***^	0.188^***^	0.188^***^	0.083	0.083	0.083
	(0.113)	(0.113)	(0.113)	(0.047)	(0.047)	(0.047)	(0.060)	(0.060)	(0.060)
10	0.300^**^	0.300^**^	0.300^**^	0.175^***^	0.175^***^	0.175^***^	0.067	0.067	0.067
	(0.115)	(0.115)	(0.115)	(0.056)	(0.056)	(0.056)	(0.049)	(0.049)	(0.049)
*R*^2^	0.151	0.141	0.160	0.050	0.139	0.140	0.060	0.108	0.111

Estimates of linear probability models explaining the likelihood of decision R by player A and decision r by player B. Standard errors (in parantheses) are clustered at the session level in the Human condition (three clusters per game matrix, six in total) and individual level in the Robot condition (40 clusters per game matrix, 80 in total) and computed using the delete-one jackknife procedure. Models 1 and 2 include a single measure of cognitive skills (a dummy set to 1 for a positive CRT score, or Raven's test score), while Model 3 combines both variables. Other independent variables include gender, game matrix and round dummies. The number of observations is N = 600 for Human and N = 800 for Robot conditions.

*/**/*** *indicate significance at the 10/5/1% level*.

We first turn to player Bs' behavior. Models 1 and 2 suggest that both the coefficient of *CRT* > 0 dummy and the coefficient of Raven's test score are positive and significant (*p* = 0.067 for *CRT* > 0 and *p* = 0.015 for Raven). In Model 3, the coefficient of Raven's test score remains highly significant (*p* = 0.014), while the coefficient of CRT becomes insignificant (*p* = 0.253). Their joint significance (*p* = 0.034) implies that cognitive skills predict the use of dominant strategy.

We now turn to player As' behavior in the Human condition. Notwithstanding the previous set of results, cognitive skills are not found to explain player As' choices. The coefficient of *CRT* > 0 dummy is insignificant (*p* = 0.226) in Model 1, and so is the coefficient of Raven's test score (*p* = 0.633) in Model 2. If we account for both, Model 3 reveals that the coefficients of both scores are neither individually (*p* = 0.226 for *CRT* > 0 and *p* = 0.550 for Raven's test score) nor jointly significant (*p* = 0.503). Finally, the behavior of player As in the Robot condition is only predicted by Raven's test score: unlike *CRT* > 0 dummy, its coefficient remains positive and highly significant across models (*p* ≤ 0.001). Unsurprisingly, the joint insignificance of both coefficients in Model 3 is also rejected (*p* = 0.003).

Altogether, the results presented in Table 6 suggest that cognitive skills predict certain components of strategic behavior: the use of dominant strategy (reflected in player Bs' behavior), as well as the ability to best respond to other player's dominant strategy (reflected in player As' behavior in the Robot condition). Moreover, in both cases Raven's test score is a more reliable predictor of behavior than CRT score. However, we also observe that Raven's test score fails to predict player As' behavior once player Bs' behavior becomes uncertain, that is once we move from Robot to Human condition. This, in turn, points toward an interplay between the degree of strategic uncertainty, behavior in the experimental games, and individual cognitive skills. Importantly, the existence of such an interplay is also supported by Figure 2 which shows that the aggregate levels of efficiency shift upwards between the Human condition and the Robot condition for the 2nd and 3rd Raven's score tertile, but not the 1st tertile.

In order to formally test this conjecture, we now look at the reaction of player As with different cognitive skills to the disappearance of strategic uncertainty. Splitting the data according to Raven's score tertile, for each of the three subsamples we compare player As' behavior in the Human condition to their behavior in the Robot condition by regressing player As' choice on the Robot dummy (set to 1 for the Robot and to 0 for the Human condition). We also include the previous set of independent variables (except for Raven's test score itself).

These results are summarized in Table 7. The coefficient of the Robot dummy captures the effect of eliminating strategic uncertainty on player As' behavior for each of the three subsamples. This suggests that only player As with high enough cognitive skills are sensitive to the uncertainty about player Bs' behavior. The behavior of players with low Raven's test score (1st tertile) is unresponsive to the degree of strategic uncertainty: the coefficient of the Robot dummy is close to zero and insignificant (*p* = 0.822). For players with medium scores (2nd tertile), we find a positive yet weakly significant effect (*p* = 0.087) which becomes amplified and highly significant for those player As whose Raven's test score belongs to the 3rd tertile of the experimental sample (*p* = 0.012).

**Table 7:** **The effect of strategic uncertainty and cognitive skills: evidence from player As' behavior in Human and Robot conditions**.

	**Raven's test score tertile**		
	**1st**	**2nd**	**3rd**
Constant	0.277^*^	0.592^***^	0.330^**^
	(0.147)	(0.065)	(0.135)
1[Robot]	0.044	0.158^*^	0.428^**^
	(0.195)	(0.090)	(0.155)
1[CRT>0]	0.002	0.038	0.016
	(0.262)	(0.066)	(0.188)
1[Male]	0.144	0.033	0.212
	(0.145)	(0.063)	(0.179)
1[Game 2]	0.034	−0.245^**^	−0.176
	(0.146)	(0.092)	(0.155)
Round dummies	Yes	Yes	Yes
*N*	480	610	310
*R*^2^	0.048	0.173	0.298

Estimates of linear probability models on decision R by player A. Standard errors (in parentheses) are clustered at the session level in the Human condition (three clusters per game matrix, six in total) and individual level in the Robot condition (40 clusters per game matrix, 80 in total) and computed using the delete-one jackknife procedure. Data from Human and Robot conditions are pooled and split into three subsamples based on Raven's test score tertiles. Other independent variables include a dummy set to 1 for a positive CRT score, as well as gender, game matrix and round dummies (omitted from the table).

*/**/*** *indicate significance at the 10/5/1% level*.

Finally, it is also worth noting that player As' reaction to the payoff scheme also varies as a function of Raven's test score. The coefficient of the Game 2 dummy is close to zero and highly insignificant in the 1st tertile regression (*p* = 0.890). Then, it becomes negative in 2nd and 3rd tertile models (although it is only statistically significant in the former with *p* = 0.012 and *p* = 0.271, respectively). This, in turn, stands in line with the previous finding that player As' willingness to play *R* increases as the safe choice *L* becomes less attractive relative to outcome (*R, r*). It also seems that the magnitude of this effect is mediated by player As' cognitive skills, although not in a monotone way.

## 4. Conclusion

This paper studies the relationship between strategic behavior and cognitive skills—cognitive reflection and fluid intelligence—in a classic 2 × 2 dominance-solvable game. Our results show that subjects with higher fluid intelligence (measured by Raven's progressive matrices test) are more likely to play dominant strategy, and also more likely to best respond to other's strategy. Furthermore, fluid intelligence predicts strategic sophistication: only those players with sufficiently high Raven's test score are found to display sensitivity to the presence of uncertainty about others' behavior. Cognitive reflection (measured by CRT), in turn, lacks the power to predict behavior in our experimental setting. We see three main conclusions that stem from these findings.

First, these results contribute to the ongoing debate on the relationship between rationality and intelligence (see Stanovich, 2009, for a critical review). For instance, Stanovich and West (2014) distinguish between two aspects of rational behavior: instrumental rationality which is understood as the “ability to take appropriate action given one's goals and beliefs,” and epistemic rationality which enables agents to hold “beliefs that are commensurate with available evidence.” In the strategic environment investigated in this paper, instrumental rationality can be associated with the ability to solve the game, while epistemic rationality—with the ability to play it with others. Our experimental data suggest an important relationship between fluid intelligence (rather than reflective thinking) and both of these facets of rationality in strategic settings. Both the ability to use dominance and iterated dominance to efficiently solve the game, as well as the responsiveness to the availability of strategic information, is found to be predicted by Raven's test score (but not by CRT score).

The second contribution is related to the experimental methodology. Despite the fact that CRT and Raven's test are both commonly used to measure cognitive skills in experimental subject pools, still very little is known about their relative performance in predicting different types of behavior. Therefore, the choice of one test over the other may happen to be at least as intuitive as evidence-based. As mentioned before, to the best of our knowledge only two experiments address this issue. Brañas-Garza et al. (2012) do so in a strategic environment (*p*-beauty contest game), while Corgnet et al. (2015a)—in a non-strategic one (individual choices on wealth distribution). Both studies find that CRT performs better than Raven's test in predicting subjects' behavior. The result of the present experiment points the to the opposite conclusion. We believe that this difference is driven by the very nature of the experimental tasks which may involve different types of cognitive effort. In our view, this issue deserves attention in future research.

Finally, although we find evidence that behaving in accordance with dominance solvability is positively correlated with cognitive skills, we also substantiate that most of the variance in individual decision making cannot be explained by such skills. Thus, exploring factors alongside cognitive skills that generate strategic behavior remains an open and important empirical question. An interesting avenue is to disentangle individual determinants, e.g., personal characteristics (such as cognitive skills) that are associated with appropriate behavior, from environmental determinants, that is, those features of the decision making environment that lead decision makers to take certain types of actions.

## 5. Author contributions

NH, NJ, SL, and AZ all contributed equally to this work. Authors are listed in an alphabetical order.

## 6. Funding

This project has received funding from JSPS-ANR bilateral research grant BECOA (ANR-11-FRJA-0002), as well as the LABEX CORTEX (ANR-11-LABX-0042) of Université de Lyon, and LABEX OSE of the Paris School of Economics (ANR-10-LABX_93-01), both within the program “Investissements d'Avenir” (ANR-11-IDEX-007) operated by the French National Research Agency (ANR). Ivan Ouss provided efficient research assistance. We thank Juergen Bracht, Colin Camerer, Guillaume Fréchette, Haoran He, Asen Ivanov, Frédéric Koessler, Rosemarie Nagel, Ariel Rubinstein, Jason F. Shogren, Jean-Marc Tallon, Antoine Terracol, and Marie Claire Villeval for their comments. NH and NJ gratefully acknowledge the *Institut Universitaire de France*. SL thanks the School of Business at the University of Western Australia for hospitality and support. A major part of this work was conducted while NH was affiliated with Aix-Marseille University (Aix-Marseille School of Economics, AMSE) and NJ was affiliated with Université de Lorraine (BETA). NH and NJ thank both institutions for their various supports.

### Conflict of interest statement

The authors declare that the research was conducted in the absence of any commercial or financial relationships that could be construed as a potential conflict of interest.

## Footnotes

^1^This class of games has been first introduced by Moulin (1986) as the *p*−beauty contest games, where *p* (often equal 2/3) stands for the target fraction of all numbers' average.

^2^See Nagel (1995) and Ho et al. (1998) for early evidence from the laboratory, Costa-Gomes and Crawford (2006) for a laboratory experiment supporting a behavioral model of bounded rationality, and Bosch-Domenech et al. (2002) for related evidence from the field.

^3^Both Camerer (2003) and Crawford (2004) consider this game as a basic example of a dominance-solvable game, and a glaring case of a mismatch between theoretical predictions and actual behavior.

^4^If the game is played sequentially (so that player A moves first), the same solution can be obtained through backward induction. Note that if *s* > *h*, the solution does not change (since *l* remains player B's weakly dominated strategy), but the outcomes are no longer Pareto-rankable. Beard and Beil (1994), Schotter et al. (1994), and Goeree and Holt (2001) find that this environment also generates important violations of standard theoretical predictions.

^5^Cognitive skills are often measured using (amongst others) the Cognitive Reflection Test (CRT, Frederick, 2005), the Raven's progressive matrices test (Raven, 2008), or both (like in this study). The details of these two measures are presented in Section 2.

^6^RMET consists of a series of photos of the area of the face involving the eyes. Subjects are asked to choose one of the four words that best describes what the person in the photo is thinking or feeling.

^7^See Hampton et al. (2008) for related evidence.

^8^Al-Ubaydli et al. (2013, in press) also report that individual cognitive skills do not predict individual willingness to reach efficient outcomes in these two game.

^9^Herein, we restrict our design to these two game matrices and do not seek to further investigate the effects of monetary incentives on both players' behavior. These effects are analyzed in detail in Jacquemet and Zylbersztejn (2014) and Hanaki et al. (2016).

^10^An English translation of the original instructions in French is provided as supplementary material.

^11^See Jacquemet and Zylbersztejn (2013) for a detailed motivation and description of this design.

^12^Absence of monetary incentives for providing corrects answers is a standard procedure for both CRT and Raven's tests. Recent evidence on both tests suggests that monetary incentives do not *per se* affect people's performance. See Brañas Garza et al. (2015) for a metastudy on the determinants of CRT scores and Eckartz et al. (2012) and Dessi and Rustichini (2015) for experimental evidence on the role of monetary incentives in Raven's test.

^13^See Raven (2008) for an overview.

^14^Model 3 suggests that these two proportions are not significantly different: testing *H*_0_ : β_1_ + β_3_ = 0 yields *p* = 0.303. The increase in the proportion of decisions *R* between Human and Robot conditions is insignificant for Game 1 (*H*_0_ : β_2_ = 0, *p* = 0.679) and significant for Game 2 (*H*_0_ : β_2_ + β_3_ = 0, *p* = 0.054).

^15^See also Frederick (2005) and Bosch-Domènech et al. (2014) for related evidence.

^16^We test the difference in proportion of a given outcome between two experimental conditions by carrying out a bootstrap proportion test that accounts for within-subject correlation, i.e., the fact that the same individual takes 10 decisions. The procedure consists of bootstrapping subjects and their corresponding decisions over all 10 rounds instead of bootstrapping decisions as independent observations (see e.g., Jacquemet et al., 2013, for a detailed description of the procedure).

^17^Given that most CRT scores in our sample are null and the higher the score, the less frequent it gets, dichotomizing the CRT score variable limits the impact of the outliers on the overall results.
