# Supplementary material for: Fluid Intelligence and Cognitive Reflection in a Strategic Environment: Evidence from Dominance-Solvable Games
Source: Front Psychol. 2016 Aug 10;7:1188. doi: 10.3389/fpsyg.2016.01188 (PMC4978737; doi:10.3389/fpsyg.2016.01188)
Supplement: Supplementary file 1 [file Image1.pdf]

English translation of the original instructions for Game 1 in French. Variations according to Human/Robot conditions appear in paragraphs preceded by **[Robot]** / **[Human]**.

You are about to take part in an experiment in which you can earn money. *The amount of your gains will depend on your decisions, as well as on decisions made by other participants.*

Before starting, we would like to ask you to answer a few standard questions (concerning your age, education, profession, ...) which will help us to get to know you better. **This information, as well as the amount of your gains from this experiment, will remain strictly confidential and anonymous.**

Please fill in the questionnaire using the interface on your computer screen, which is divided into three parts:

- In the <u>top</u> section, you will find information that might help you in making decisions.
- In the <u>middle</u> section, you will submit your decisions by clicking on a relevant button.
- In the <u>bottom</u> section, you will see all your decisions and gains from previous rounds of the experiment.

Thank you.

The experiment

[Human] The experiment consists of several identical rounds. In each round, participants are divided into groups of two. Each pair consists of one player A and one player B. You will be randomly assigned to your role — player A or player B — at the beginning of the experiment, and retain it throughout the experimental session. A message on your computer screen will inform you about your role. **Your role will remain unchanged throughout the entire experiment.**

[Robot] The experiment consists of several identical rounds. In each round, participants are divided into groups of two. Each pair consists of one player A and one player B. Player B is an automated player whose behavior is described below. Throughout the experiment, **you will thus play as player A, and the computer will play as player B.**

What happens in each round

[Human] At the beginning of each round, participants are divided into pairs: if you are player A, then a player B is randomly selected to complete your pair; analogously, if you are player B, then a player A is randomly selected to complete your pair. Your pair will **change after each round**, and two participants in opposite roles **will interact at most once during the experiment.**

Each round consists of 4 stages.

Stage 1. At the beginning, a participant is randomly matched to complete your group.

Stage 2. Player A chooses between L and R by clicking on a relevant button on his/her computer screen.

Stage 3. Player B chooses between l and r by clicking on a relevant button on his/her computer screen.

Stage 4. End of the round and each player is informed about his/her earnings:

- If **player A chose** L, then **regardless of player B's decision**:
  - ▶ Player A earns 8.50 €in this round;
  - ▶ Player B earns 3 €in this round;
- If **player A chose** R then:
  - if **player B chose** l:
    - ▶ Player A earns 6.50 €in this round;
    - ▶ Player B earns 4.75 €in this round;
  - if **player B chose** r:
    - ▶ Player A earns 10 €in this round;
    - ▶ Player B earns 5 €in this round;

At the end of each round, a message on your computer screen will inform you either that a new round is about to start, or that the experiment is over.

[Robot] In this experiment the computer chooses r at each round, without exception.

Payment of your earnings

At the end of the experiment, **one round is picked at random**. Each participant receives a sum in EUR corresponding to the amount he/she earned in this round, plus a bonus of 5 €for completing the experiment. Payments are made individually and in cash.

For obvious reasons, **you are not allowed to talk during the experiment**. Participants who violate this rule will be excluded from the experiment and all payments. It is crucial that you fully understand the rules of this experiment. Should you have any questions, please raise your hand, a staff member will answer you in private.

Thank you for your participation.
